# Design, manufacture, and control of a low-cost positive airway pressure device

**DOI:** 10.1016/j.ohx.2024.e00559

**Published:** 2024-07-14

**Authors:** Jordan F. Hill, Samuel Jackson, Mia Uluilelata, Samrath Sood, Jaimey A. Clifton, Ella F.S. Guy, J. Geoffrey Chase

**Affiliations:** Department of Mechanical Engineering, University of Canterbury, Christchurch, New Zealand

**Keywords:** CPAP, Respiratory device, Open-source, Multipurpose equitable positive airway pressure

## Abstract

Current positive airway pressure devices cost NZ$800-$2500, posing a financial barrier for the estimated 1 billion individuals worldwide with sleep apnea and those researching respiratory diseases. Increasing diagnoses and research interest in the area necessitate a low-cost, easily accessible alternative. Thus, the mePAP, a high-quality, multipurpose, low-cost (∼NZ$250) positive airway pressure device, was designed and prototyped specifically for respiratory disease research, particularly for sleep apnea. The mePAP allows user customization and provides researchers with an affordable tool for testing positive airway pressure algorithms. Unlike typical commercial devices, the mePAP offers adaptability with open-source data collection and easily modifiable software for implementing and analysing different control and diagnostic algorithms. It features three control modes: constant; bilevel; and automatic; and provides pressures from 4 to 20 cmH2O, controlled via a phone app through Wi-Fi, with a mini-sensor added at the mask for increased accuracy. Validation tests showed the mePAP’s performance is comparable to a gold-standard Fisher & Paykel device, with extremely similar output pressures. The mePAP’s low cost enhances accessibility and equity, allowing researchers to test ventilation algorithms for sleep apnea and other respiratory conditions, with all data openly available for analysis. Its adaptability and multiple applications increase its usability and usefulness across various research and clinical settings.

## Graphical abstract

**Figure:**
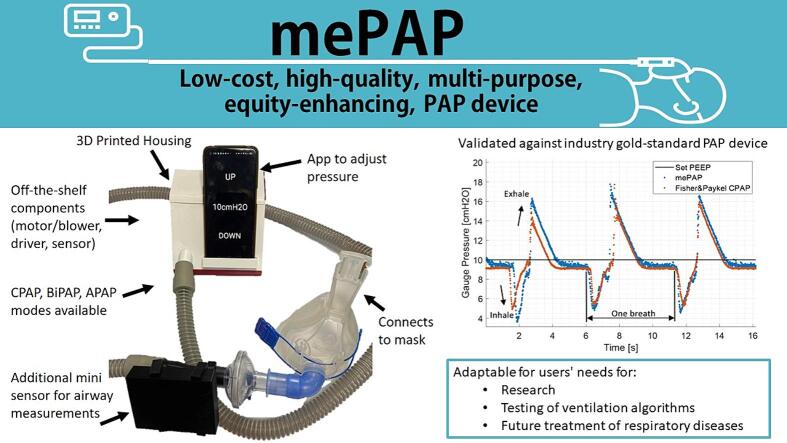

Specifications table

**Table:** 

Hardware name	mePAP (Multipurpose Equitable Positive Airway Pressure Device)
Subject area	• Biomedical Engineering • Mechanical/Mechatronics Engineering • Open source and low-cost alternatives to existing infrastructure
Hardware type	• Mechanical engineering and materials science • Respiratory device • Measuring physical properties and in-lab sensors
Closest commercial analog	CPAP devices are commercially available from Fisher & Paykel, Resmed etc. However, they are expensive with no open-source data or ability to modify detection algorithms. The additional mini-sensor at the airway of the mePAP is not present in any commercial products.
Open source license	Creative Commons Attribution 4.0 International license
Cost of hardware	∼ NZ$250 for base device (NZ$400 if including additional mini-sensor)
Source file repository	https://doi.org/10.17632/ng2k62crcg.2

**Table:** 

Nomenclature	
mePAP	Multipurpose equitable positive airway pressure
NZ$	New Zealand Dollar
PAP	Positive airway pressure
CPAP	Continuous positive airway pressure
BiPAP	Bilevel positive airway pressure
APAP	Automatic positive airway pressure
PEEP	Positive end-expiratory pressure
PCB	Printed circuit board
PWM	Pulse width modulation
PLA	Polylactic acid
PID	Proportional, integral, differential
CAD	Computer-aided design
STL	Stereolithography

## 1 Hardware in context

The mePAP is a multipurpose, equity-enhancing (low-cost), PAP device for research, with potential future treatment of respiratory diseases, in particular sleep apnea with additions and modifications. Sleep apnea is a prevalent respiratory illness, in which blocked airways cause repeated stopping and starting of breathing during sleep, which has long-term negative impacts on health and early mortality [1], [2], [3], [4], [5]. Over 1 billion individuals worldwide are estimated to have sleep apnea, with 1 in 5 adults having mild symptoms [6], [7]. Treatment for sleep apnea is achieved through PAP therapy, which maintains a set PEEP to keep airways open [8], [9].

Current PAP devices cost NZ$800-$2500, creating a large financial barrier for patients, especially as lower socioeconomic users are more susceptible to sleep apnea [10], [11]. The cost also hinders respiratory researchers from accessing PAP devices for testing. With data not openly accessible, further costs are required to collect and analyse data from current PAP devices. Increasing respiratory disease diagnoses and research interests necessitate a low-cost, easily accessible PAP alternative. Therefore, a mePAP device is thus an alternative low-cost, high-quality, equity-enhancing device for PAP research. It is made of basic off-the-shelf components situated in a 3D-printed housing. The device works standalone as a CPAP, with an additional ‘mini-sensor’ housing produced and placed at the airway directly before the mask, for added control to provide BiPAP and APAP delivery modes. Usage is simplified with operation through a phone app with Wi-fi control, which is readily extensible to Bluetooth, any near-field communications mode, or even USB cable. The mePAP aims to provide researchers with a foundational PAP device to build on. Its primary intended use is for research, offering open-source data collection for analysis and a device for testing new treatment and ventilation algorithms.

Current PAP devices are produced by companies such as Fisher & Paykel (Auckland, New Zealand) and Resmed (San Diego, CA, USA). Limited information is provided on their specifications or control algorithms, along with all use data withheld from users and/or not stored. With many extra features like humidification, CPAPs can be costly, with BiPAP and APAP devices adding further costs for more advanced and comfortable care.

CPAP devices have one constant pressure set for the entire use period, being the most basic PAP device. BiPAP devices have two set operating pressures: a higher inspiration setting (e.g. 10 cmH2O); and a lower expiration setting (e.g. 8 cmH2O) to reduce excess pressure being applied, increasing sleep efficiency while preventing overdistension of the lung tissue. APAP devices are the most complex, with automatic PEEP adjustments performed on a breath-by-breath basis from additional inline and auditory sensor measurements to detect periods without breathing [18]. Adjustments between clinician-set boundaries of 4–20 cmH2O are performed through algorithms based on the detection of inspiratory flow limitation, snoring, apnea events, and obstructive pressure in device sensor measurements [12]. These algorithms are normally based on average male breathing patterns, with limited information provided to researchers on how they function, impeding evaluation and usefulness outside of the set demographic.

PAP devices commonly provide only one treatment type, either CPAP, BiPAP, or APAP compared to the multipurpose mePAP which can provide all three main modalities. With only the base essential components, the mePAP can be easily adapted to best suit the required set-up. The housing can be modified along with algorithms for breath and apnea detection in BiPAP and APAP mode. This customisation allows for new software to be tested with results collected and analysed with just one device.

The mePAP was developed by reducing a basic PAP device to its essential elements and reconstructing it using inexpensive, off-the-shelf components. Any parts not necessary for the device to function were removed. Focus was then placed on making the mePAP adaptable and easily modifiable to suit the widest range of user needs. Finally, making the device simple to use with all data able to be collected drove the development of the phone app control and additional mini-sensor. The originality and uniqueness of the mePAP lie in its low cost, open access to data, additional airway mini-sensor, and the ability to modify any part of the device, specifically its control and detection algorithms. Hence, these areas were focused on during its development.

## 2 Hardware description

**Fig. 1:**
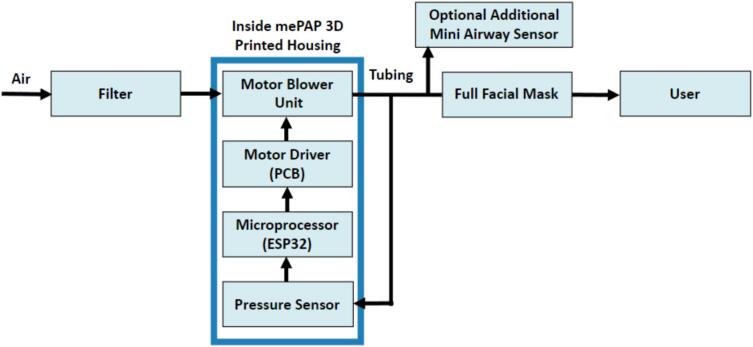
mePAP base components and connection layout.

The mePAP consists of a simple 3D-printed housing, with a 12 V brushless DC motor blower unit, a motor driver (PCB), and a pressure sensor encased inside. Some current devices include or can attach, a humidification unit to warm the air to increase comfort. The devices are fully functional without this addition, and thus it was not included to keep costs low without affecting the useability. However, it could be added if required. As seen in Fig. 1, air enters the mePAP housing through a filter, before entering the motor blower unit. The blower is controlled by a motor driver, which receives signals from the ESP32 microprocessor. The microprocessor processes the pressure readings from the pressure sensor connected to the tubing. Air leaves the blower unit at the set pressure and travels along the tubing to a full facial mask which is attached to a user or connected to a mechanical lung.

Pressure levels from 4 to 20 cmH_2_O are comfortably achieved, at 12 V and 3 A. Pressure to 30 cmH_2_O is possible with increased fluctuations. However, patients are rarely prescribed positive pressure greater than 15 cmH_2_O so testing is unlikely needed above this threshold [13]. The mePAP is controlled through an ergonomic mobile phone or tablet app. Open access to breathing data throughout use is currently withheld on many PAP devices, preventing analysis of the device.

The device is created for easy modification to best suit a user’s needs. Adaptations can be made to the housing, algorithms, and components. Higher accuracy motor/blowers, motor drivers, and pressure sensors can be used for a more precise system or even lower-cost components with the same specifications selected to decrease costs.

The additional mini-sensor at the airway provides another level of accuracy with closed-loop feedback control implemented to ensure the pressure the subject receives at their airway is as close as possible to the value set. It also provides further measurements for collection, storage, and analysis. The mini-sensor sends airway pressure and flow data to the mePAP’s microprocessor through Wi-Fi. This unit has been designed to fit at the end of the tubing and connect to the mask with ease, being small and lightweight to minimise disruptions to the user. Like the base, the algorithm and design of the mini-sensor can be adapted for different sensors and detection methods. No current PAP devices to date connect to or utilize external sensor systems, which highlights a further and unique advantage of the mePAP.

Users should be aware the current design is a prototype and has not been proven to meet the requirements to act as a regulatory-approved medical device. In its current state, the mePAP is useable only in a research setting. It can provide pressure from 4 to 20 cmH2O in three modes with minimal pressure error and bias, along with the ability to adapt algorithms as required. The device has been used for PAP algorithm evaluation and in two clinical research trials in place of a commercial PAP device to have open access to all data. The mePAP is designed to be used by researchers, who can modify the device to suit their needs. In future, with modifications and standard-based regulatory trials, the mePAP could potentially be used for PAP treatment. Main modifications include printing of parts with biocompatible resin for long-term use, noise reduction, and further safety features to meet medical device regulations, as the current trials demonstrate the ability to deliver accurate ventilation modes.

The mePAP provides users with:

• A low-cost, open-source alternative to a commercial PAP device for research use
• Ability to test CPAP, BiPAP, and APAP models and algorithms
• Unrestricted access to data with non-invasive data collection
• Simple to use with an ergonomic control phone app
• High level of customization dependent on user requirements

### 2.1 Design files

#### 2.1.1 CAD files

The physical design was performed in SolidWorks 2022 [14]. The mePAP housing consisting of the base, front panel, and lid is provided along with the tube for connection from the motor blower to the long tubing. The mini-sensor housing box and venturi for measuring flow at the airway are provided. This is not essential for running the CPAP. However, for more accurate results and BiPAP and APAP performance, it should be included. The base design can be changed to fit users' aesthetic desires. SolidWorks part files (SLDPRT) are provided for user adaptation.

#### 2.1.2 3D printing

STL files for 3D printing all components, including the housing, venturi, and mini-sensor are provided for printing parts as is. STL files were imported into PrusaSlicer for GCODE production before printing on a Prusa 3D printer. All SolidWorks part files are provided as an STL for direct printing.

#### 2.1.3 Electronics

PCBs were designed in Altium [15]. Local library files are included for the schematic and footprints used. The PCB was designed with connectors to house an ESP32, with a simple bipolar transistor to convert the power from 5 V to 3 V3. The same PCB is used for both the base mePAP and the additional mini-sensor. A pressure sensor PCB is also required for the main housing and mini-sensor. These two PCBs could be combined into one at the user’s preference. PCBs were manufactured by JLCPCB and populated in the Surface Mount Laboratory at the University of Canterbury.

#### 2.1.4 Software and firmware

The code for driving and controlling the motor for CPAP, BiPAP, and APAP modes along with the mini-sensor is included as C files in Visual Studio Code [16]. MATLAB code for data collection and plotting of results is also provided [17]. The phone app for mePAP control via Wi-Fi is provided as a txt file to be copied into the DroidScript Android app. This app can be adapted for different phone types with the colours and layout easily changed to fit the user’s needs. Bluetooth can also be used to control the device rather than Wi-Fi.

## 3 Design files summary

All the design files are available at https://doi.org/10.17632/ng2k62crcg.2. Table 1 outlines file locations.

**Table 1:** Design file summary.

**Design file name**	**File type**	**Open source license**	**Location of the file**
Base	CAD (SLDPRT) and STL files	CC BY 4.0	Device, Hardware, 3D printed Components, Main Housing
Lid	CAD (SLDPRT) and STL files	CC BY 4.0	Device, Hardware, 3D printed Components, Main Housing
Tube	CAD (SLDPRT) and STL files	CC BY 4.0	Device, Hardware, 3D printed Components, Main Housing
Screen Phone	CAD (SLDPRT) and STL files	CC BY 4.0	Device, Hardware, 3D printed Components, Main Housing
Box Assembly	CAD (SLDPRT) and STL files	CC BY 4.0	Device, Hardware, 3D printed Components, Main Housing
Attachment Clip	CAD (SLDPRT) and STL files	CC BY 4.0	Device, Hardware, 3D printed Components, Mini-Sensor Housing
Flow Venturi	CAD (SLDPRT) and STL files	CC BY 4.0	Device, Hardware, 3D printed Components, Mini-Sensor Housing
PCB Base	CAD (SLDPRT) and STL files	CC BY 4.0	Device, Hardware, 3D printed Components, Mini-Sensor Housing
PCB Top	CAD (SLDPRT) and STL files	CC BY 4.0	Device, Hardware, 3D printed Components, Mini-Sensor Housing
PCB ESP32	Altium files	CC BY 4.0	Device, Hardware, PCBs
PCB Pressure Sensor	Altium files	CC BY 4.0	Device, Hardware, PCBs
CPAP	C file	CC BY 4.0	Device, Code, ESP32 Code
BiPAP	C file	CC BY 4.0	Device, Code, ESP32 Code
APAP	C file	CC BY 4.0	Device, Code, ESP32 Code
Mini-Sensor	C file	CC BY 4.0	Device, Code, ESP32 Code
App Code	Txt file	CC BY 4.0	Device, Code, App
Data Collection	MATLAB file	CC BY 4.0	Device, Code, Matlab Collection

## 4 Bill of materials summary

The completed bill of materials is attached in an Excel spreadsheet, available at https://doi.org/10.17632/ng2k62crcg.2. This spreadsheet outlines components and their cost for the base mePAP (∼NZ$250) and the additional mini-sensor (∼NZ$150).

## 5 Build instructions

**Fig. 2:**
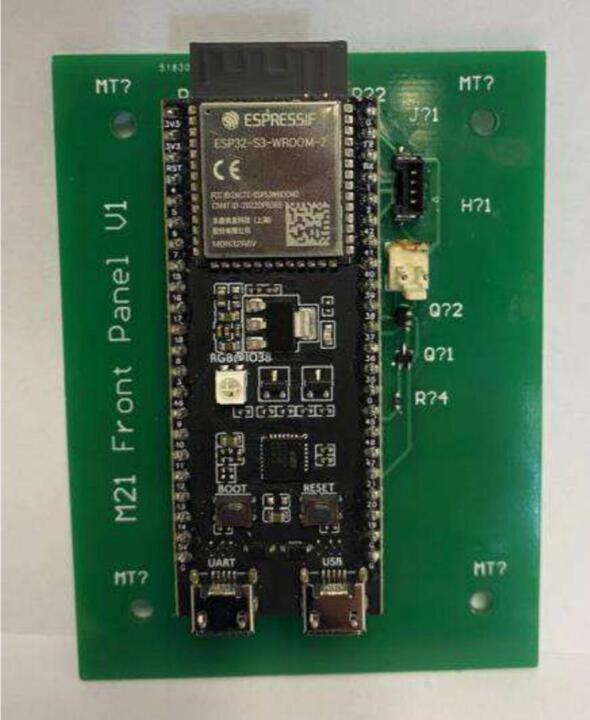
Populated PCB ESP32.

To construct the device, the instructions are as specified:

1. Place an order for the PCBs and appropriate parts required for population *(PCB ESP32 and PCB Pressure Sensor).* With manufacturing and shipping times this step is often slow so should be completed first. The motor and driver should also be purchased at this time.
2. Populate the PCBs in accordance with the Altium project files. To populate the *PCB ESP32* first solder on the capacitors, resistors, and cable connectors (listed in BoM). Solder female socket headers into the through holes for the ESP32, before mounting it. The populated board with ESP32 attached is seen in Fig. 2. The *pressure sensor PCB* requires resistors, the pressure sensor, connectors, and a capacitor to be soldered as seen in Fig. 3.

**Fig. 3:**
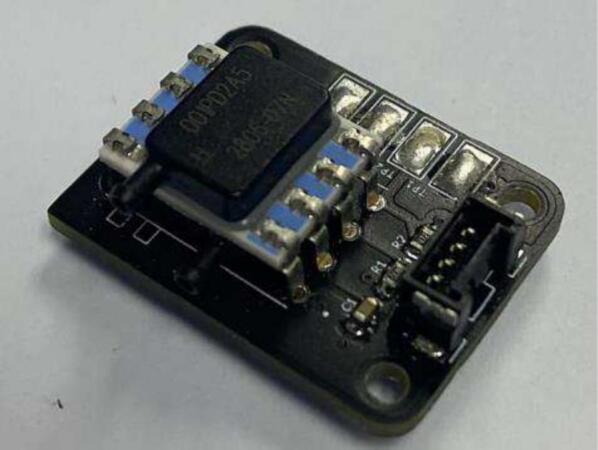
Populated PCB Pressure Sensor.
3. Print all 3D printed parts (base, lid, screen phone, tube) in the device, hardware, 3D printed parts folder with appropriate layer height (eg. 0.015 mm). The orientation of the 3D printed parts is shown in Fig. 4a and b with support material indicated in green. Parts were printed on an Original Prusa MK4 3D printer using PLA. Support material was removed with pliers. Parts should be altered before printing if required.

**Fig. 4:**
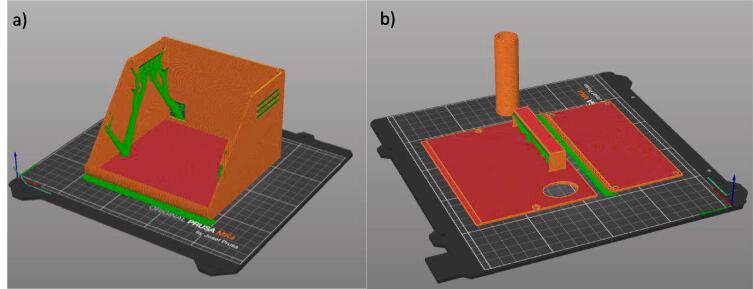
a. Print orientation for the base component (orange/red) with support in green. 4b: Print orientation for the lid, screen, and tube. (For interpretation of the references to colour in this figure legend, the reader is referred to the web version of this article.)
4. The 3D printed tube has pressure sensor port guide holes which are drilled out using a 1.6 mm drill bit. Using a knife, the pressure sensor port copper tube was cut to a 5 mm length and inserted into the tube hole before being glued in place with superglue. The bottom of the tube must be flush with the interior surface of the printed tube for accurate pressure readings. Use minimal glue to prevent any blockages. Flexible silicone tubing connects the pressure sensor port to the port tube as shown in Fig. 5. This tube length can be adapted to fit the desired layout of the mePAP.

**Fig. 5:**
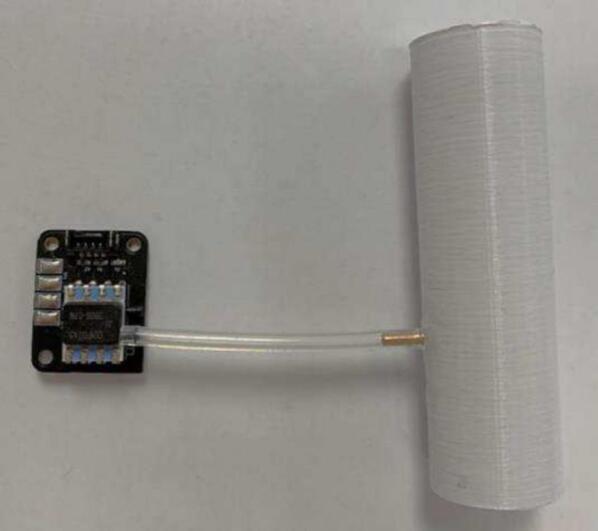
Tube connected to the pressure sensor.
5. The motor is inserted into the larger opening end of the tube until it hits the small rim inside. A tight connection is required to prevent any large pressure losses. The motor driver and *PCB pressure sensor* are then connected to the *PCB ESP32* via wire connections. A USB C cable is inserted into the UART port of the ESP32, to complete the connection of all parts as shown in Fig. 6.

**Fig. 6:**
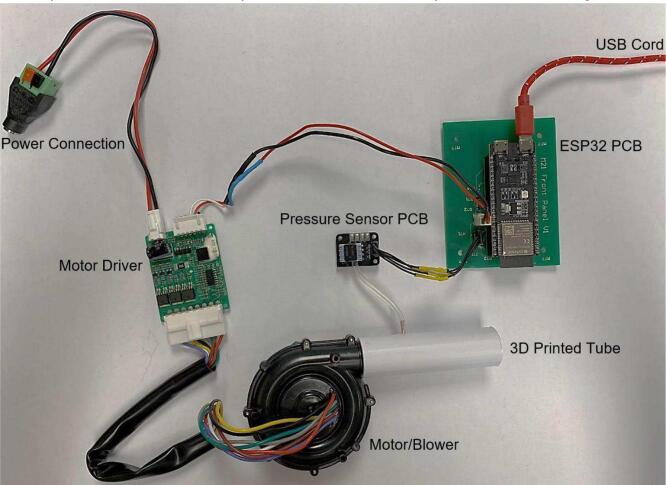
Exploded view of mePAP connected components.
6. A layer of hard 10 mm thick polyurethane foam was cut using a craft knife to the size of the mePAP base (130 mm x 140 mm). This was placed flush on the base of the mePAP. A layer of acoustic foam the same size was cut to sit on top. A hole for the motor and venturi was cut out of the acoustic foam for noise reduction and to allow the components to sit tightly. The motor, driver, and pressure sensor were then placed inside with the PCB and motor cords threaded out the small hole in the back of the base. A second layer of acoustic foam was placed on top of the components before the ESP32 PCB was inserted on top. A single layer of acoustic foam was then placed on top of the ESP32 PCB for further noise reduction. More or different foam can be added to further increase noise acoustics, with the component’s placement not crucial as long as they remain connected.
7. Six M3 bolts were then screwed to attach the front screen phone and lid to the base to enclose the box for a fully assembled mePAP (Fig. 7).

**Fig. 7:**
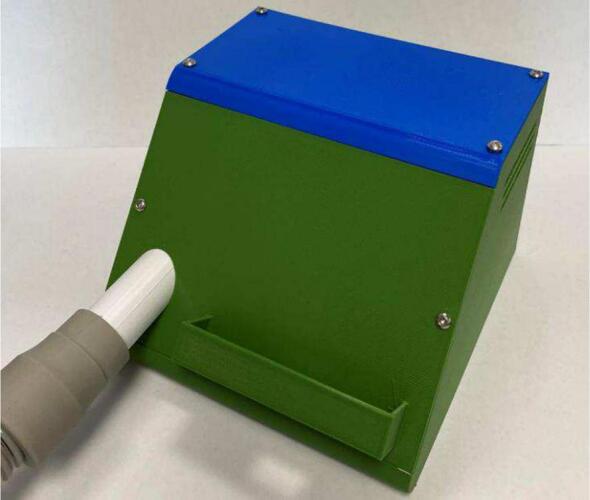
Assembled mePAP.
8. The mePAP can be connected to a benchtop power supply set to 12 V and 3 A for use. A push knob connector as seen in Fig. 6 can also be added for connection into a 12 V, 5 A switching adapter laptop charger for mains use.

### 5.1 Additional Mini-Sensor

If the additional mini sensor is used for BiPAP and APAP modes, the following construction is also required. For convenience, the mini-sensor uses the same two PCBs (*PCB ESP32* and *PCB pressure sensor)* as the mePAP. The mini-sensor venturi is used for flow measurements at the airway, with the min-sensor housing placed directly before the filter and mask.

1. Populate another *PCB ESP32* and *PCB pressure sensor*, as outlined in step 2 above.
2. 3D print the mini housing, attachment clips, and venturi files (Device, Hardware, 3D printed Components, Mini-Sensor Housing Parts Folder) as seen in Fig. 8.

**Fig. 8:**
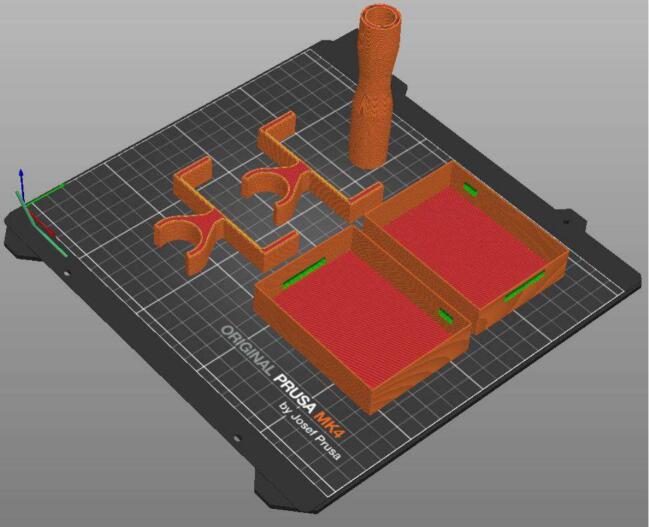
Print orientation for the mini-sensor housing parts (orange/red) with support in green. (For interpretation of the references to colour in this figure legend, the reader is referred to the web version of this article.)
3. Repeat step 4 above, adding the pressure sensor ports to the venturi as seen in Fig. 9. The venturi in the mini-sensor is used to measure flow at the airway for inhale, exhale, and no flow detection. Two holes, one at the throat of the venturi and one before the slope are required to be tapped to connect both pressure sensor ports for measurement of the differential pressure to determine flow. The throat venturi tube connects to the bottom port of the pressure sensor, and the other to the top pressure sensor port.

**Fig. 9:**
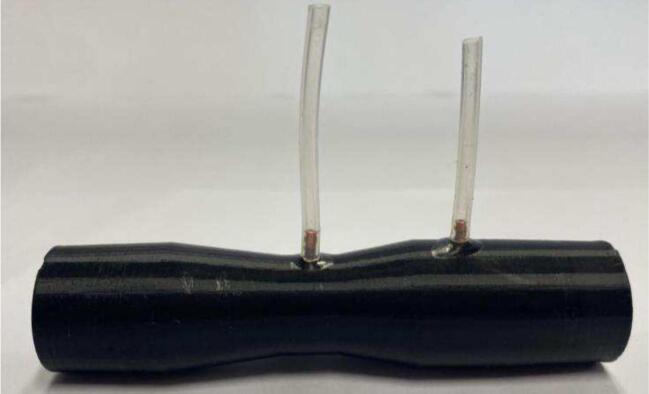
Venturi with taped holes and silicone tubes attached.
4. Insert the *PCB ESP32* and *PCB pressure sensor* into the 3D-printed housing. Slide the circular ends of the printed attachment clips onto the venturi. Clip the sensor housing into the other side of the attachment clips. Connect the end of the venturi with the tapped pressure port to the mePAP tubing and the other end to the filter connected to the mask. If required, the filter can be removed for testing, with the venturi connecting directly to the face mask. This completes the entire mePAP system as seen in Fig. 10.

**Fig. 10:**
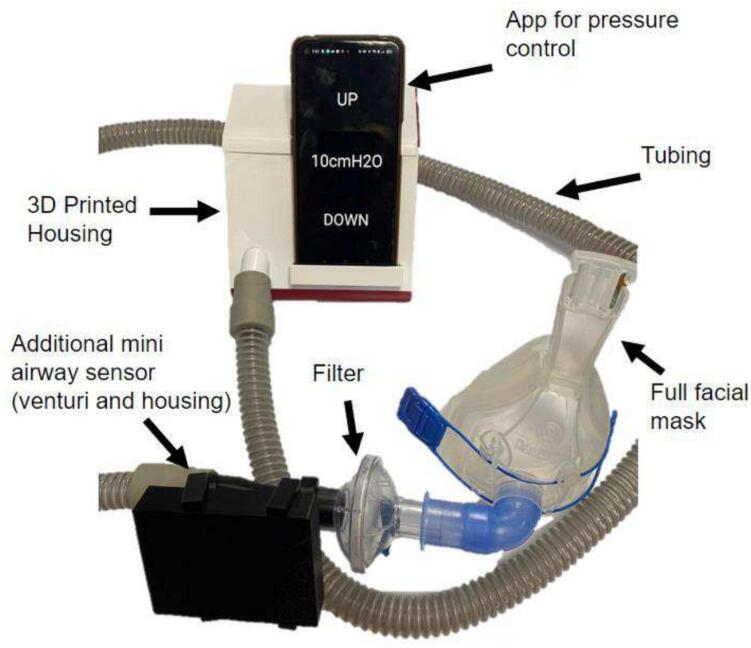
Completed mePAP with phone app, mask, and additional mini-sensor connected to a filter which connects to the mask.

The mini-sensor is not required for the base CPAP but allows for additional airway data to be collected and for flow to be detected for BiPAP and APAP modes.

## 6 Operation instructions

Once constructed the operational use of the mePAP is performed as follows:

1. Connect the respiratory circuitry for the desired use to the end of the tube. If the additional sensor is used this sits between the end of the tube and mask (Fig. 10). Most commonly a filter and a full-face mask are used.
2. Decide what mode the mePAP will be used in: CPAP; BiPAP; or APAP; and upload the corresponding code to the ESP32. For BiPAP and APAP with the additional mini-sensor, *BiPAP* or *APAP* code is uploaded to the base sensor ESP32, and *mini-sensor* code to the ESP32 in the mini-sensor housing. Connect the ESP32 to a computer for uploading files ensuring they have built correctly in PowerShell.
3. Download DroidScript and open the app file (*App Code*) on an Android device. Connect the phone's Wi-Fi to the ESP32 station and ensure a connection has been established to allow for sending of pressure data. Currently ‘mywifissid’ is the station name. This can be changed to the user's liking in the code under Wi-Fi name. The phone must be connected to the ESP32 Wi-Fi station (base ESP32) for the app to run.
4. In CPAP mode, open the phone app and set the desired pressure by tapping the up or down buttons as seen in Fig. 11. Pressure increases in steps of 0.5 cmH_2_O. Tapping the set pressure number (8 cmH_2_O in Fig. 11) will reset the mePAP to the base pressure of 4 cmH_2_O. This base pressure can be adjusted in the CPAP code. In BiPAP or APAP mode, the app is not required as pressures are set by clinicians and so are built into the code. Set pressures and step sizes can be changed by adapting the code.

**Fig. 11:**
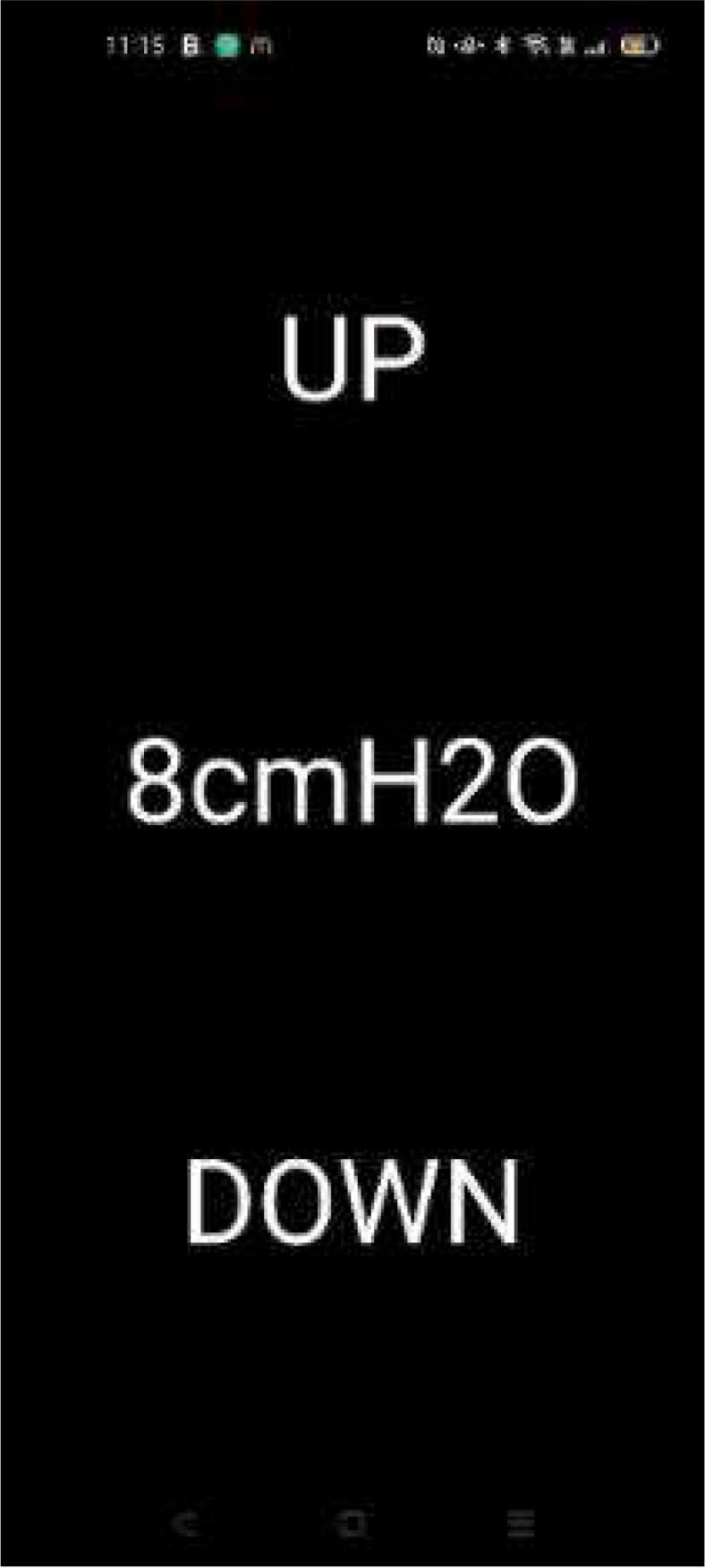
mePAP phone control app user interface.
5. To collect data, open *data_collection* on MATLAB. Specify the subject number, test type, and recording length in the initial lines of code (yellow in Fig. 12). Adjust the COM port number as required. The serial monitor on the code runner used for the C files must be closed for MATLAB to record data. The code can then be started by clicking run. It will automatically stop and save the data once the length of the trial is complete. Data is saved as a.mat file for analysis on MATLAB or the user's chosen program. The.mat file contents depend on the collection site of the base or airway mini-sensor. If data is collected from the mePAP base, gauge pressure is provided, compared to flow or gauge pressure measurements at the mini-sensor depending on the set mode and user requirements. Data can be collected from both the mini-sensor and the mePAP simultaneously.

**Fig. 12:**
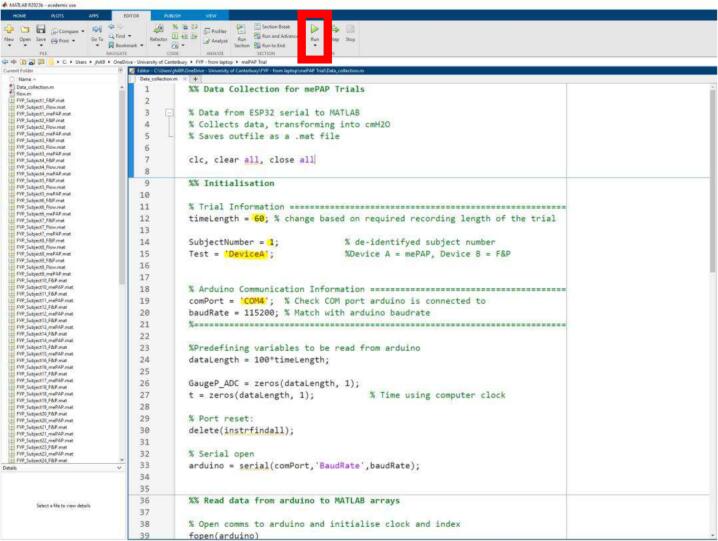
MATLAB data collection code. Highlighted yellow are code lines that require changing. The code is run by clicking run (red box). (For interpretation of the references to colour in this figure legend, the reader is referred to the web version of this article.)
6. These results can then be analysed with the plotting functions as required.

### 6.1 Operation modes

**Fig. 13:**
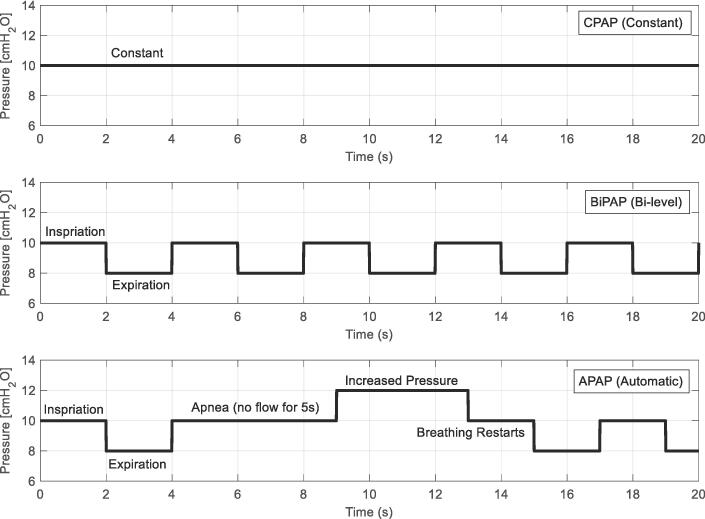
Desired (ideal) PAP pressure plots.

The mePAP can be run in three modes: CPAP; BiPAP; and APAP. Fig. 13 shows the desired pressure plots for each mode. These are ideal, with disruptions from breathing affecting the measured pressure. The mePAP’s expected behaviour is as close to the ideal plots as possible while accounting for breathing disturbances. CPAP provides one constant set pressure. This pressure can be changed through the phone app, or manually adjusted in the code. Inhale and exhale will initially decrease then increase the measured pressure, creating spikes, before reducing back to the constant set pressure. BiPAP changes between two set pressures (BiPAP_Inhalation and BiPAP_exhalation), increasing the ease of breathing out for the user. The mePAP acts like the desired plot, but with rounded edges as the measured pressure does not change instantly. APAP works identically to BiPAP with the added feature of apnea detection. If no flow has been detected for more than 5 s since the last inhale, the pressure is increased until breathing restarts and BiPAP continues as normal. Again, the mePAP behaves similarly to the desired plot, with discrepancies due to natural breathing.

### 6.2 Software operation

**Fig. 14:**
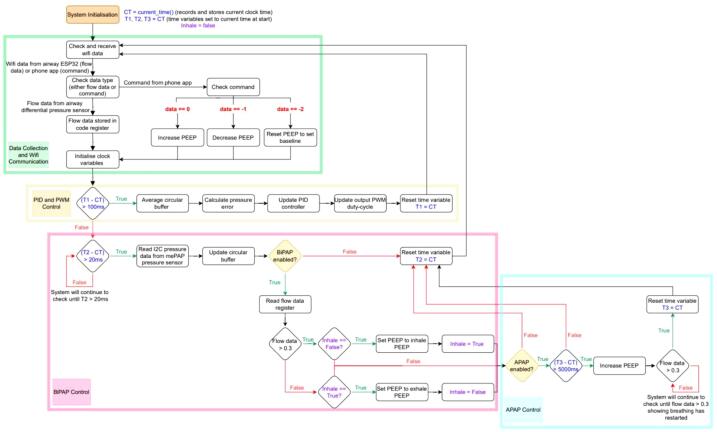
Flowchart of mePAP closed loop motor control.

The mePAP has been designed for testing different algorithms, especially in BiPAP and APAP mode. Any set pressures, step amounts, and detection methods can all be adapted to suit the user. Fig. 14 shows a high-level flow chart of the provided software algorithm for closed-loop motor control of the mePAP. The control is broken into four main program blocks: data collection and Wi-Fi communication (green); PID and PWM control (yellow); BiPAP (pink); and APAP (blue).

Variables in blue represent time values used by the program schedular to run tasks. Initially, all the time variables (T1, T2, and T3) are set to the current clock time (CT). As the program runs, the elapsed time (current time compared to initialisation time, eg. T1-CT) is checked against different criteria to determine what control loop to perform. Once an elapsed time check has been met, the variables are reset for the loop to repeat. Flow data comparisons of > 0.3, are differential pressure thresholds, which are compared against the data in the Wi-Fi register from the mini-sensor measurements. These thresholds can be adapted as required. Purple variables show inhale or exhale, where Inhale = False, assigns the inhale variable to false, and Inhale == False, compares the two, checking if inhale is set to false or not.

The green program block controls the data collection from Wi-Fi communication between the base ESP32, mini-sensor ESP32, and the phone app. With the mePAP device having input from multiple devices, the program determines the type based on whether the transmitted value is > 0 (mini-sensor airway data). If not, the commands 0, −1 and −2 are reserved for pressure input commands from the mePAP phone control app. These command values are arbitrary, with numbers selected as they never appear in the transmitted data values from the mini-sensor. Commands from the phone app increase (0), decrease (−1) or reset (−2) the set PEEP. Commands are set to increase/decrease PEEP in 0.5 cmH_2_O steps, with reset to a baseline of 4 cmH_2_O. Flow data from the mini-sensor at the airway is collected and stored in a register for comparisons in later control blocks.

The yellow program loop updates the PWM output of the software at a period of 100 ms. This sub-program uses an averaged I2C pressure value to calculate the estimated error in the system and updates the PID controller to adjust for the estimated error. The estimated error is calculated as the difference between the set PEEP from the phone command and the average measured buffer pressure. The PID controller finally updates the output PWM which is sent to the motor driver, to ensure the delivered pressure matches the set PEEP.

The pink control loop reads and stores the I2C pressure data into a circular buffer and compares the present values to determine the presence of BiPAP and APAP activation features. If BiPAP is enabled, a flow data threshold of 0.3 must be exceeded to trigger the increase of set PEEP to 10 cmH_2_O for patient inhalation. Flow data is collected from the differential pressure sensor in the additional airway mini-sensor. If the current flow data is less than this threshold, PEEP is set to or remains at the lower setting for patient exhalation (8 cmH_2_O).

Finally in the blue control box, if APAP is enabled, the elapsed time from the beginning of the previous detected inhale to the current time is checked. If this time exceeds 5 s, APAP is triggered with the PEEP set above the upper BiPAP setting to 12 cmH_2_O. The mePAP device will remain at this raised pressure until flow data indicates an inhalation feature (flow data > 0.3) causing the device to switch back to BiPAP mode. All set pressures, thresholds, and timings can be adapted by the user in the provided code. Specifically, APAP can be adapted, with more complex measures added to further improve apnea detection.

### 6.3 Health and safety considerations

If built according to instructions, and used as intended, there are minimal safety hazards for using the mePAP. Users should take care when building the device to ensure wires are connected properly, and PCB components have been soldered correctly. The mePAP is designed to operate on a 12 V power supply. Using voltages outside the recommended range may cause the device to behave unexpectedly. The mePAP is provided as a foundation for building a usable PAP device. In its current state, despite testing, it is not a certified or regulated medical device. If testing on individuals, set appropriate pressure levels to prevent lung distention from excessive pressure.

## 7 Validation and characterisation

The mePAP performs as a CPAP, BiPAP, or APAP device at pressure limits of 4–20 cmH_2_O. Pressure sensors used were validated against a TSI4000 Flow meter (4000 Series Analog and Digital Flow Meter, TSI, Shoreview, MN, USA) against pressure and flows generated by an industry-standard Fisher & Paykel SleepStyle CPAP (Auckland, NZ). The mePAP was then also validated through comparison to the Fisher & Paykel CPAP device with three main tests: 1) Benchtop testing; 2) Simulated breathing on a mechanical lung; and 3) Subject breathing from a clinical trial on 40 healthy individuals.

The mePAP is designed to function comparatively to industry-standard PAP devices. The key differences between the mePAP and the selected comparison device of the Fisher & Paykel SleepStyle CPAP are outlined in Table 2. The mePAPs reduced cost, multi-operational modes, additional airway sensor, phone app control, open access to data, and ability to test detection algorithms make it unique from the Fisher & Paykel CPAP. A humidifier system to warm the provided air could be added to the mePAP if required, along with therapy hours and mask leakage data added to the phone app to provide the same features as the Fisher & Paykel CPAP.

**Table 2:** Key differences between mePAP and Fisher & Paykel SleepStyle CPAP.

	**mePAP**	**Fisher & Paykel SleepStyle CPAP**
**Cost**	NZ$250 (CPAP) or NZ$400 (BiPAP, APAP)	NZ$800–2500 depending on model
**Operation Modes**	CPAP, BiPAP, APAP	CPAP
**Airway Sensor**	Additional mini-sensor at airway	No external airway sensor
**Additional Components**	No additional components, only basic essential parts	Humidifier
**Control**	Controlled via phone app	Controlled via buttons on the device
**Data**	Open access to all data	Restricted access to data
**Therapy Data on Device**	No current data implemented on the device	Therapy hours and mask leakage available
**Algorithm Adaptability**	Ability to change detection algorithms for testing	No ability to change detection algorithms
**Mask/filter**	Any filter or full facial mask with standard sizing	Any filter or full facial mask with standard sizing
**Housing Size (w** × **d** × **h)**	130 × 140 × 90 mm	160 × 150 × 130 mm

### 7.1 Benchtop testing

**Fig. 15:**
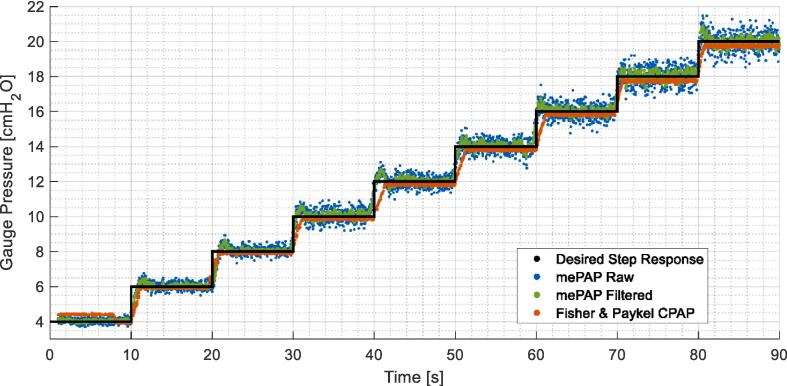
Increasing pressure of the mePAP and Fisher & Paykel CPAP when connected to the stationary mechanical lung.

**Fig. 16:**
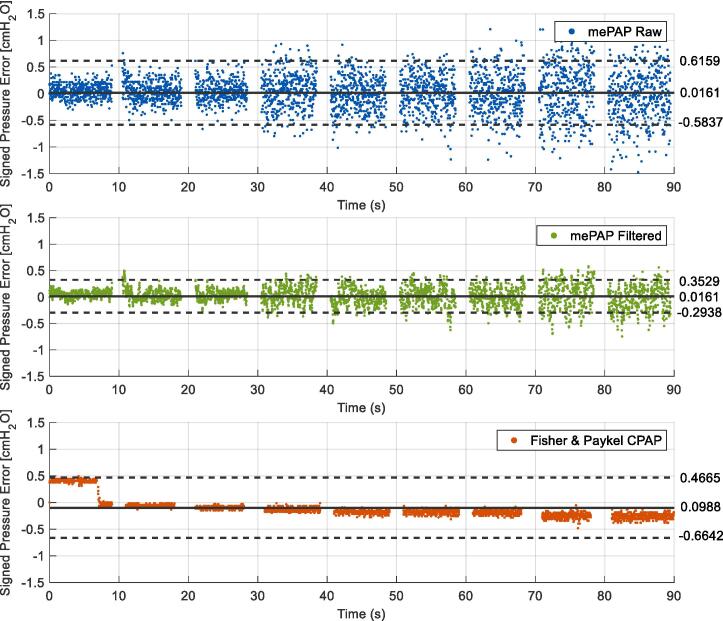
Signed pressure error over time excluding rise time, as pressure increases by 2 cmH_2_O every 10 s with mean (solid black line), and limits of agreement (dashed lines at ± 1.96 std) displayed.

**Fig. 17:**
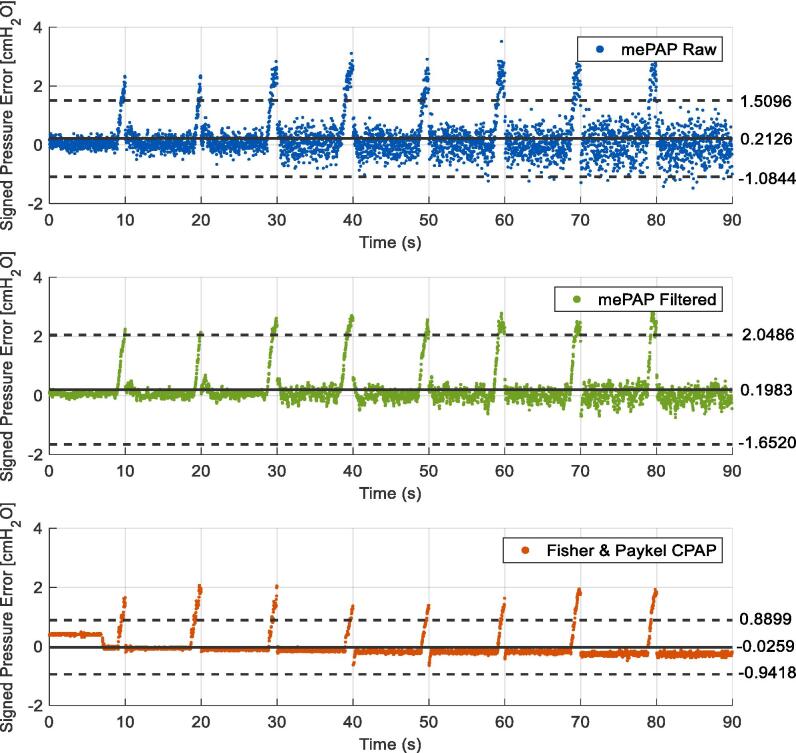
Signed pressure error over time with rise time included, as pressure increases by 2 cmH_2_O every 10 s with mean (solid black line), and limits of agreement (dashed lines at ± 1.96 std) displayed.

Early benchtop testing to deliver a set constant pressure showed fluctuations in the data as seen in Fig. 15. Testing consisted of a 90-second test starting at 4 cmH_2_O, with the pressure increased by 2 cmH_2_O every 10 s until 20 cmH_2_O was reached. This test was repeated five times with results consistent across all trials. The mePAP closely follows the step response, with fluctuations increasing as the set pressure increases. When filtered with a five-point moving average filter, the measured and set PEEP correspond more closely, with the rise time matching Fisher & Paykel. Filtering reduces overshoot and fluctuations as seen in the signed pressure error over time excluding and including rise time at pressure changes (Fig. 16, Fig. 17 respectively). The signed pressure error is the measured pressure minus the set pressure. In agreement with Fig. 15, increased pressure increases fluctuations with a larger error spread determined at larger set pressures.

Fig. 16 shows the mePAP has reduced bias compared to the Fisher & Paykel CPAP when rise times are excluded with both the raw and filtered data having a mean of 0.0161 cmH_2_O compared to 0.0988 cmH_2_O. When rise time is included the larger overshoot when the pressure changes skews the result as seen in Fig. 17. Set pressures ideally change instantly, but the motor has limitations for how quickly it can increase the pressure. Hence a larger pressure error is seen when rise time is included with higher mean values. As the Fisher & Paykel device, under delivers compared to the set pressure, including the larger rise time pressures brings the mean closer to zero.

The 95 % confidence interval is ± 1.96 standard deviation. Points outside this range show a large difference between the set pressure and the measured pressure. However, Fig. 16, Fig. 17 show the measured pressure is outside only when the pressure is increased, or from fluctuations at high pressure. With a maximum of 6.18 % of the measured pressures outside the range, the majority of points lie within the confidence interval with a uniform spread, suggesting the set and measured pressures are similar.

Table 3 shows both the mean and median pressure are larger (increased bias) when the rise time is present as expected. The mePAP interquartile range is situated around zero showing minimal bias, compared to Fisher & Paykel whose range sits just below zero. Excluding the rise time the mePAP has a median error of 0 cmH_2_O directly matching the set pressure, compared to Fisher & Paykel who deliver 0.1 cmH_2_O less. Ranges are small (all less than 0.4552), with a strong cluster in the middle 50 % of measured pressures. The filtered pressure has a tighter range and fewer points outside the confidence interval. The mePAP raw and filtered pressure errors match or exceed the Fisher & Paykel, validating the quality of the device. Where values differ is due to the increased fluctuations seen in the mePAP. Due to the low-cost motor, these fluctuations are induced by torque ripple resulting from a limited number of pole pairs and are unable to be removed without an alternative motor. Therefore, the validation with simulated breathing on a mechanical lung and clinical trial ensured the mePAP was comparable to an industry device with the fluctuations present.

**Table 3:** Signed pressure error mean, median and interquartile range (cmH_2_O) for benchtop testing.

	**mePAP Raw**	**mePAP Filtered**	**Fisher & Paykel CPAP**
**Rise Time Excluded**			
Mean	0.0161	0.0161	−0.0259
Median	0	0	−0.1149
Interquartile range	[−0.1011, 0.1671]	[−0.0483, 0.1039]	[−0.1915, 0]
**Rise Time Included**			
Mean	0.2126	0.1983	−0.0259
Median	0.0767	0.0613	−0.1410
Interquartile range	[−0.1241, 0.3311]	[−0.0555, 0.1943]	[−0.2099, −0.0628]

### 7.2 Simulated breathing on mechanical lung

The mePAP was validated with a Mechanical Lung Test providing a fully controlled bench test with repeatable breaths. A Michigan Instruments Training and Testing Mechanical Lung (Michigan, USA) was used to validate the mePAP with ‘breaths’ of known and controlled compliance and tidal volume. The lung was set to a compliance of 0.05 L/cmH2O to simulate a normal adult’s lung [18].

Breathing was simulated using the mechanical lung to determine if the device could control positive pressure with externally induced changes in lung pressure due to breathing-induced changes in lung volume. Tidal volume was increased to 0.5L over 1 s to simulate inspiration. The lung then was allowed to passively collapse over 3 s to simulate passive exhalation. This process was repeated for 1 min with the mePAP and the Fisher & Paykel CPAP. Both setups were tested five times at each PEEP level between 4–20 cmH_2_O.

**Fig. 18:**
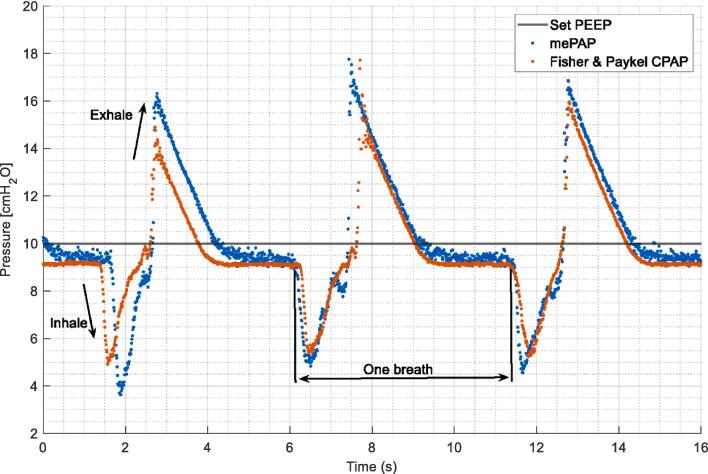
Simulated breathing response for each device on the mechanical lung at 10 cmH2O.

All recorded data from this validation testing was collected from the mini-sensor unit at the airway for a more accurate measurement of pressure delivered to the patient airway. Data from the mechanical lung provides sharp inhalation and exhalation peaks as seen in Fig. 18. Pressure rise and settling times are comparable, with the mePAP reaching a marginally higher pressure at peak exhalation. Both devices settle to a lower constant pressure than the set 10 cmH2O with the mePAP at 9.5 cmH2O, and Fisher & Paykel at 9 cmH2O.

The pressure profile during breathing on the mechanical lung is formed from the pressure differential created between the ‘lung’ and the ‘airway opening’ as it draws flow in by lung expansion and forces it out during recoil and contraction. A CPAP device is designed to maintain a constant airway pressure as seen in Fig. 13. However, in reality, CPAP therapy cannot and does not, instantaneously compensate for pressure fluctuations during breathing, as seen in Fig. 16. Thus, the degree of fluctuation can indicate the responsiveness of the CPAP device, with lower fluctuations indicating a higher responsiveness. Comparing the mePAP to the Fisher & Paykel device, both have similar maximum pressure fluctuations (Fig. 16), validating the mePAP’s performance compared to industry-standard equipment. Finally, the pressure fluctuations during breathing in Fig. 18 are lower than when simply held constant in initial bench testing in Fig. 15, Fig. 16, Fig. 17.

### 7.3 Clinical trial

The mePAP was also validated with a clinical trial on 40 healthy patients [19]. Each trial consisted of 5 min of breathing on the mePAP with the pressure increased from a PEEP of 4cmH2O to 14cmH2O in 2cmH2O increments every 30 s, with 15 s changing time between to account for the rise time. The same test was repeated on the Fisher & Paykel Device, with an extra blindfolded 3-minute switch test at the conclusion, to see if subjects could feel a difference between the two devices. All breaths at all set pressure levels were comparable between the two devices, with results matching those seen on the mechanical lung (Fig. 18). The fluctuations present in the mePAP during bench testing do not affect the quality or comfort for users, with the mePAP having a mean comfort rating of 6.36 out of 10 compared to Fisher & Paykel at 5.92.

**Fig. 19:**
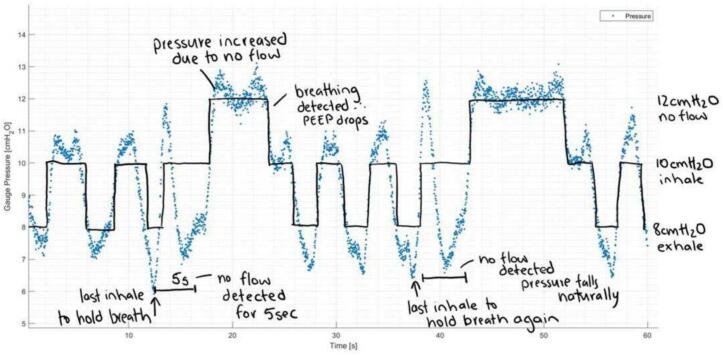
BiPAP and APAP implementation. Measured pressure data in blue and ideal PEEP in black. (For interpretation of the references to colour in this figure legend, the reader is referred to the web version of this article.)

BiPAP and APAP modes were validated through subject breathing with pressure readings and flow recorded. Tests were performed on 4 individuals. Each test ran for 2 min and was repeated 5 times. BiPAP is set for 10 cmH_2_O during inhalation and 8 cmH_2_O for exhalation. These set pressures can be changed by adapting the BiPAP_Inhalation and BiPAP_exhalation values in the provided code (*BiPAP*). APAP was tested by subjects holding their breath, with the pressure set to increase to 12 cmH_2_O if no flow had been detected for more than 5 s after the previous inhale. This metric can also be adjusted, with time increased or decreased depending on the user's normal breathing rate. Fig. 19 shows the inhale and exhale patterns sitting at the set pressures before increasing to 12 cmH_2_O when flow stops. This pressure response matches the desired response in Fig. 13, validating all modes of the device. No commercial BiPAP or APAP device was available for comparison. However, as the device performed consistently as expected, it was deemed acceptable.

### 7.4 Noise testing

Noise testing was conducted through the Decibel X app (SkyPaw Co. Ltd, Vietnam). In testing, the average CPAP device produces approximately 30–40 dB of noise, equivalent to the sound of leaves rustling or a whisper [20]. Both the mePAP and gold standard Fisher & Paykel CPAP were tested at the most common pressure of 10 cmH_2_O, with the Decibel detector phone approximately 0.5 m away to simulate the approximate distance between the device and a person sleeping. The Fisher & Paykel device over 1 min of testing averaged 42.7 db noise, compared to the mePAP at 51.5 db. This value is still in the normal sleep range, but further efforts to reduce noise are required. Notably, the mePAP is significantly smaller than the Fisher & Paykel CPAP, which allows more room for foam and noise reduction. When the mePAP was placed into a larger box of similar size to a CPAP device with additional foam the average noise was 44 db, nearly equivalent to Fisher & Paykel. Thus, quieter therapy can be achieved from the mePAP, which will require better/more foam insulation, a larger and/or more acoustically designed box, or/and a more expensive and quieter motor.

Characterisation of hardware performance:

• mePAP operates with a 12 V external power supply with a current limit of 3 A.
• mePAP can provide pressure between 4–20 cmH_2_O in three different settings of CPAP, BiPAP, and APAP.
• mePAP comes with an additional sensor for more accurate flow detection at the airway to increase precision in provided pressure.
• The motor is designed for a lifetime of 10,000 h. However, this has not been tested for the mePAP.
• mePAP pressure, flow, and pressure error mean, median and ranges are comparable to industry standard Fisher & Paykel CPAP.

### 7.5 Potential user changes

The mePAP has been designed with adaptability at its forefront, for use in testing different algorithms and detection methods across all three PAP types. This provides the user with many options for potential changes including, but not limited to:

• Additional or different sensors for detection of pure flow, sound, movement, or any metric that may be of interest to the user
• Different housing shapes and designs, including a larger box or further noise reduction
• Alternative components, namely a more expensive motor or driver for increased accuracy
• A new phone app or control system
• Connection into a closed-loop system for real-time control monitoring
• Bluetooth connection rather than Wi-Fi
• Further conversion to mains power supply
• Reversal of therapy to provide mechanical ventilation
• Different control algorithms
• More than one additional mini-sensor
• Additional data collection methods

With the key components of the motor, sensor, and control, everything else can be adapted easily to best suit the users’ requirements. This customizability is not present with current PAP machines, with the mePAP providing the ability to try new metrics and analyse them with its open-source data collection.

## 8 Ethics statements

Ethical consent for the trials using this hardware was granted by the Human Ethics Committee at the University of Canterbury (Ref: HREC 2023/46/LR). Subjects were given a written and verbal explanation of the test protocol before signing a consent form. Subjects were made aware they could inform a researcher if they wished to withdraw from the testing at any point and testing would be terminated immediately with their data deleted.

## CRediT authorship contribution statement

**Jordan F. Hill:** Writing – review & editing, Writing – original draft, Validation, Software, Methodology, Investigation, Conceptualization. **Samuel Jackson:** Validation, Software, Methodology, Investigation. **Mia Uluilelata:** Validation, Software, Methodology, Investigation, Conceptualization. **Samrath Sood:** Validation, Software, Methodology, Investigation, Conceptualization. **Jaimey A. Clifton:** Writing – review & editing, Supervision. **Ella F.S. Guy:** Writing – review & editing, Supervision. **J. Geoffrey Chase:** Writing – review & editing, Supervision.

## Declaration of competing interest

The authors declare that they have no known competing financial interests or personal relationships that could have appeared to influence the work reported in this paper.
